# Intracranial epidermoid cysts: benign entities with malignant behavior: experience with 36 cases

**DOI:** 10.1038/s41598-023-33617-x

**Published:** 2023-04-20

**Authors:** Maria Mihaela Pop, Dragos Bouros, Artsiom Klimko, Ioan Alexandru Florian, Ioan Stefan Florian

**Affiliations:** 1 Department of Neurosurgery, Iuliu Hatieganu University of Medicine and Pharmacy, Cluj-Napoca, Romania; 2 Clinic of Neurosurgery, Cluj County Emergency Clinical Hospital, Cluj Napoca, Romania; 3 Laboratory of Molecular Neuro-Oncology, Department of Neurology, University Hospital Zurich, Zurich, Switzerland

**Keywords:** Neurology, Oncology, Risk factors

## Abstract

Intracranial epidermoid cysts are benign slow-growing ectodermal inclusions that account for less than 1% of all intracranial tumors. We retrospectively reviewed 36 such cases to evaluate the demographic characteristics, clinical manifestations, anatomical distribution, surgical management, and treatment outcome of these tumors. Additionally, we sought to identify the relationship between median or paramedian cistern tumor localization and clinical parameters, such as recurrence risk, hospitalization duration, and postoperative complication rates. The most frequently observed neurological symptoms were transient headaches (77.8%), dizziness (36.1%), CN VII palsy (19.4%), CN VIII hearing difficulty (19.4%) and cerebellar signs (19.4%). The most common surgical approaches included retrosigmoid (36.1%), subfrontal (19.4%) and telovelar (19.4%) approaches; gross total resection was feasible in 83.3% of cases. The postoperative complication rate was 38.9%. Tumors were more frequently found in the paramedian cisterns (47.2%), followed by the median line cisterns (41.6%). Multivariate analysis revealed that postoperative hydrocephalus and age < 40 years were prognostic factors for tumor recurrence. Median-like tumor location was a risk factor for the presence of symptomatic hydrocephalus both preoperatively and postoperatively, increasing the likelihood of protracted hospitalization (> 10 days). Despite their benign histopathological nature, these tumors have an important clinical resonance, with a high rate of postoperative complications and a degree of recurrence amplified by younger age and hydrocephalus.

## Introduction

Epidermoid cysts are benign congenital inclusion cysts that in rare cases may occur intracranially, accounting for approximately 0.3–1.8% of primary intracranial tumors^1^. Although called cysts, these “pearly” tumors are solid and are encased by a collagenous capsule that can infiltrate along CSF (cerebrospinal fluid)-filled spaces^2,3^. Tumor growth is driven by the division of the stratified squamous epithelium lining its cavity, meaning that the cyst contents are largely composed of acellular keratin debris and cholesterol inclusions. According to the literature, liquefaction of the cyst contents is associated with infection or loss of vascularity^4^. Approximately 90% of intracranial epidermoid cysts are located in the intradural compartment, although they can also occur extradurally in the intradiploic space of the frontal, parietal, temporal, and occipital bones^5–7^. Epidermoid cysts are most commonly found in the cerebellopontine angle (approximately 40% of all cases), the parasellar region (30%), and the fourth ventricle (5–18%) and less commonly in the middle cranial fossa, diploe or spinal canal^1,8,9^. The role of arachnoid compartments and structures cannot be neglected. The limitations of epidermoid formation into a specific cistern are demonstrated by the similar aspects of the epidermoid cyst at least for a period of time. Once the epidermoid cyst grows, the membranes and other arachnoidian structures, along with the neurovascular structures, will guide the further expansion of the tumor, and as a consequence, extension in different subarachnoid compartments is not an exception^10^.

Despite their slow growth, these tumors have a strong propensity to adhere to critical neurovascular structures and cause impingement secondary to the mass effect, causing significant morbidity and neurologic impairment^3,11^. The disease course is classic for an extended asymptomatic period, until the tumor capsules begin to impinge on a cranial nerve or eloquent brain tissue, resulting in a rapidly evolving deficit. Magnetic resonance imaging (MRI) is the investigation of choice, and diffusion-weighted imaging (DWI) is the most helpful sequence for differential diagnosis and follow-up^12^.

Surgical resection is at the forefront of patient management, especially because there is currently no concrete chemotherapy or targeted therapy available for this tumor type^13^. Gross total resection is critical to minimize the risk of recurrence^14^, as in some studies, incomplete removal of the capsule was associated with recurrence rates as high as 93%^15^.

The main aim of the present study was to evaluate the demographic characteristics, clinical manifestations, surgical management and clinical outcomes of patients with intracranial epidermoid tumors. To our knowledge, this is the first study to report such information in a Romanian population. Additionally, we sought to identify the relationship between median or paramedian cistern tumor localization and clinical parameters, such as recurrence risk, hospitalization duration, and postoperative complication rates.

## Methods

All methods were carried out in accordance with the current neurosurgical guidelines available. An observational retrospective study was performed on 36 patients admitted to the Department of Neurosurgery, Cluj-Napoca Country Emergency Hospital during the period 2012–2021. Patients enrolled in this study had a pathologically confirmed diagnosis of an intracranial epidermoid cyst that was surgically resected. All adult patients as well as the guardians of pediatric patients gave their informed consent for both the intervention and the participation within this study. Regarding Figs. 2 and 3 in the manuscript, we obtained consent from the patients to publish the images in an open-access online publication, keeping their personal data anonymized. No experimental protocols involved human or animal subjects or tissue samples. All operations were performed by the same neurosurgeon.

Patients were then stratified based on the localization of the tumor to the median, paramedian, or outside cisterns. Median cisterns included the fourth ventricle, cisterna magna, interpeduncular, pericallosal, suprasellar, chiasmatic, quadrigeminal, and cerebellomedullary cisterns. Paramedian cisterns were defined as the cerebellopontine, ambiens, carotid and sylvian cisterns.

Further inclusion criteria included patients who presented at the recommended periodic check-ups and who had a postoperative follow-up of at least 6 months. As this was an observational study, the protocol was exempted from approval by the Ethics Committee of the “Iuliu Hatieganu” University of Medicine and Pharmacy.

Microscopic resection was performed on all patients. Gross total resection (GTR) was considered if the lesion was completely removed along with its capsule and no residual tumor was seen on 3-month postoperative MRI (DWI sequences); it was termed subtotal resection (STR) if the entire tumor was removed but part of the capsule was left behind or if only some part of the tumor and capsule was excised. The amount of tumor removed was ascertained by verifying operative notes and follow‑up imaging. Brain CT was performed within a day of surgery in all patients post-excision to look for any bleeding in the tumor bed and any other surgery‑related complications. All patients were followed‑up post‑surgery in the neurosurgery outpatient service at our institute.

In accordance with the clinic's protocol, after the primary surgical intervention, the patients were evaluated by MRI imaging at 3 months, 6 months, 1 year and then yearly postoperatively. Tumor recurrence was assessed by DWI sequences that demonstrated diffusion restriction at the time of recurrence in the area of tumor resection and the cisterns in the immediate vicinity. If the MRI imaging controls showed tumor recurrence and the patient was clinically symptomatic, they were subjected to surgical reintervention. The exception was the asymptomatic patients who were monitored periodically with MRI imaging at 6-month intervals, some of whom became candidates for surgical intervention at the time of symptomatology appearance. The other cases did not translate into a need for resurgery as the patients continued to be asymptomatic. In the case of patients undergoing surgical reintervention, postoperative scarring distorted the normal anatomy and obliterated the dissection planes. Recurrent epidermoid cysts become more adherent to neighboring critical neurovascular structures, thus making further resection a high-risk procedure. The outcome of patients was characterized by a worsening of preoperative neurological deficits. After discharge, follow-up was performed according to the clinic's protocol.

### Data analysis

All the data from the study were analyzed using IBM SPSS Statistics 25 and illustrated using Microsoft Office Excel/Word 2013. Qualitative variables were written as counts or percentages and were tested using Fisher’s exact tests. Z tests with Bonferroni correction were used to further detail the results obtained in the contingency tables.

### Ethical approval

This retrospective study involving human participants was in accordance with the ethical standards of the institutional and national research committee and with the 1964 Helsinki Declaration and its later amendments or comparable ethical standards. The Iuliu Hatieganu University of Medicine and Pharmacy, Cluj-Napoca, Research Ethics Committee has confirmed that no ethical approval is needed.

## Results

The study group included 36 patients aged between 1 and 73 years with a median age of 40.4. In our series, epidermoid cysts showed a slight male predilection (females—44.4%, males—55.6%). The average period of hospitalization was 11.64 days (between 1 and 2 weeks), with a median of 10 days. Most patients’ cysts were located in the infratentorial region (47.2%) with a predilection for the cerebellopontine angle (38.9%). According to the anatomical distribution of epidermoid cysts, in our study, 38.9% of cases were located at the level of the cerebellopontine angle, 19.4% of cases were located at the level of the IVth ventricle, 13.9% of cases were located at the temporal region, 11.1% of cases were located at the level of the frontal lobe, 8.3% of cases were located at the level of the sellar and parasellar region, and 8.3% of cases were located in other areas, such as the orbital area, the parietal region or the pontine cistern. The Glasgow Coma Scale (GCS) at admission was 15 points in 34 patients and 14 points in 2 patients. Most patients reported an insidious onset of symptoms (77.8%).

**Figure 1:**
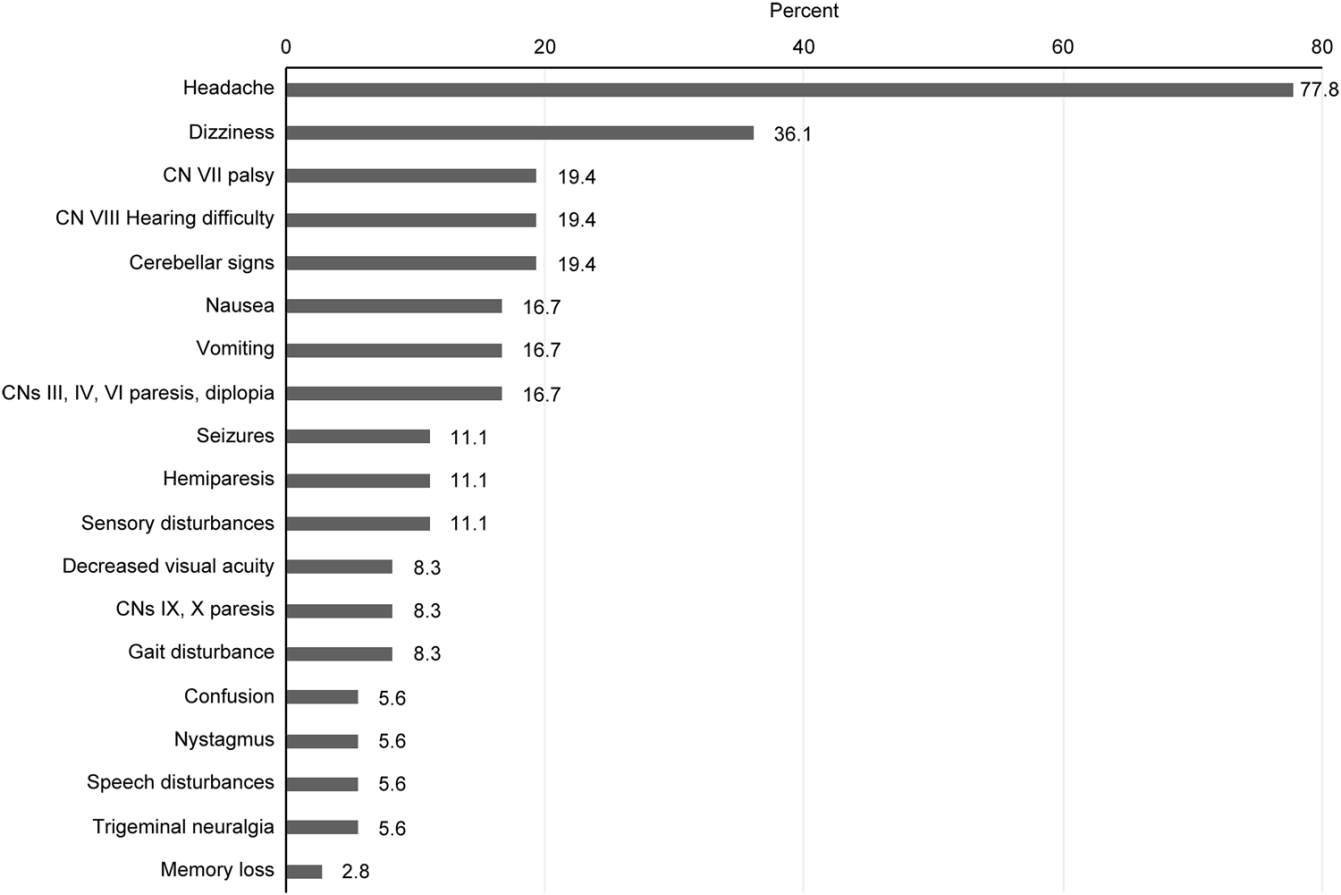
Distribution of the patients stratified by symptomatology.

The most frequently observed neurological symptoms are presented in Fig. 1 and included transient headaches (77.8%), dizziness (36.1%), CN (cranial nerve) VII palsy (19.4%), CN VIII hearing difficulty (19.4%) and cerebellar signs (19.4%).

Hydrocephalus was present in 16.7% and 19.4% of patients pre- and postoperatively, respectively. In a small subset of patients (16.7%), hydrocephalus failed pharmacologic management and required placement of a shunt, while one patient experienced transient hydrocephalus that responded to conservative management. In all patients with postoperative communicating hydrocephalus, a lumbar drain was placed for approximately 4–5 days to drain any fibrosis-inducing substances or blood clots and promote new CSF formation. If no resolution of hydrocephalus was seen, permanent CSF diversion procedures were considered. As all 6 patients had communicating hydrocephalus, a ventriculoperitoneal shunt was proposed in all cases. Of the 83.3% of patients who benefited from total resection, 5 patients developed postoperative hydrocephalus. Of the 16.7% of patients who benefited from subtotal resection, 2 patients developed postoperative hydrocephalus. In our series, we had 6 patients with hydrocephalus at presentation. Two patients presented with triventricular hydrocephalus and obstruction seen on imaging suggestive of noncommunicating hydrocephalus (obstructive mechanism). Four patients presented with tetraventricular hydrocephalus and no obvious obstruction seen on imaging suggestive of communicating hydrocephalus. In cases with communicating hydrocephalus, the proposed mechanism involves chemical meningitis, subsequent inflammation and a granulomatous reaction in the arachnoid villi with impaired CSF absorption. However, subclinical spillage of cyst contents can occur with no evident manifestation of meningism. Additionally, intricate mechanisms with both obstructive and reduced absorption are possible. Gross total resection was performed in all six cases. Of these six patients, four continued to have persistent hydrocephalus after surgery, and two patients had resolved hydrocephalus. In our series, we included a total of seven patients with postoperative hydrocephalus. Apart from the four cases discussed above, three de novo hydrocephalus cases were observed with no obstructive component.

The overall postoperative complication rate was 38.9% and included persistent hydrocephalus (6 cases), aseptic chemical meningitis (2 cases), CN VII and VIII paresis (1 case), pseudomeningocele (1 case), dysarthria (1 case), acute subdural hematoma and seizure (1 case), transient hydrocephalus and subdural hygroma (1 case) and pulmonary embolus (1 case). In our study, 8 of the patients had a duration of symptoms between 1 and 4 years; the overall postoperative complication rate in this subset of patients was 25% (2 cases) and included aseptic chemical meningitis (1 case) and pulmonary embolus (1 case). Ten of the patients had a duration of symptoms under 1 month; the overall postoperative complication rate in this subset of patients was 70% (7 cases) and included persistent hydrocephalus (4 cases), pseudomeningocele (1 case), dysarthria (1 case), acute subdural hematoma and seizure (1 case). Precipitation of symptoms (under 1 month) was associated with a higher rate of postoperative complications (p = 0.026); no association was found between long-standing symptomatic patients (more than 1 year) and the rate of postoperative complications (p = 0.44).

**Figure 2:**
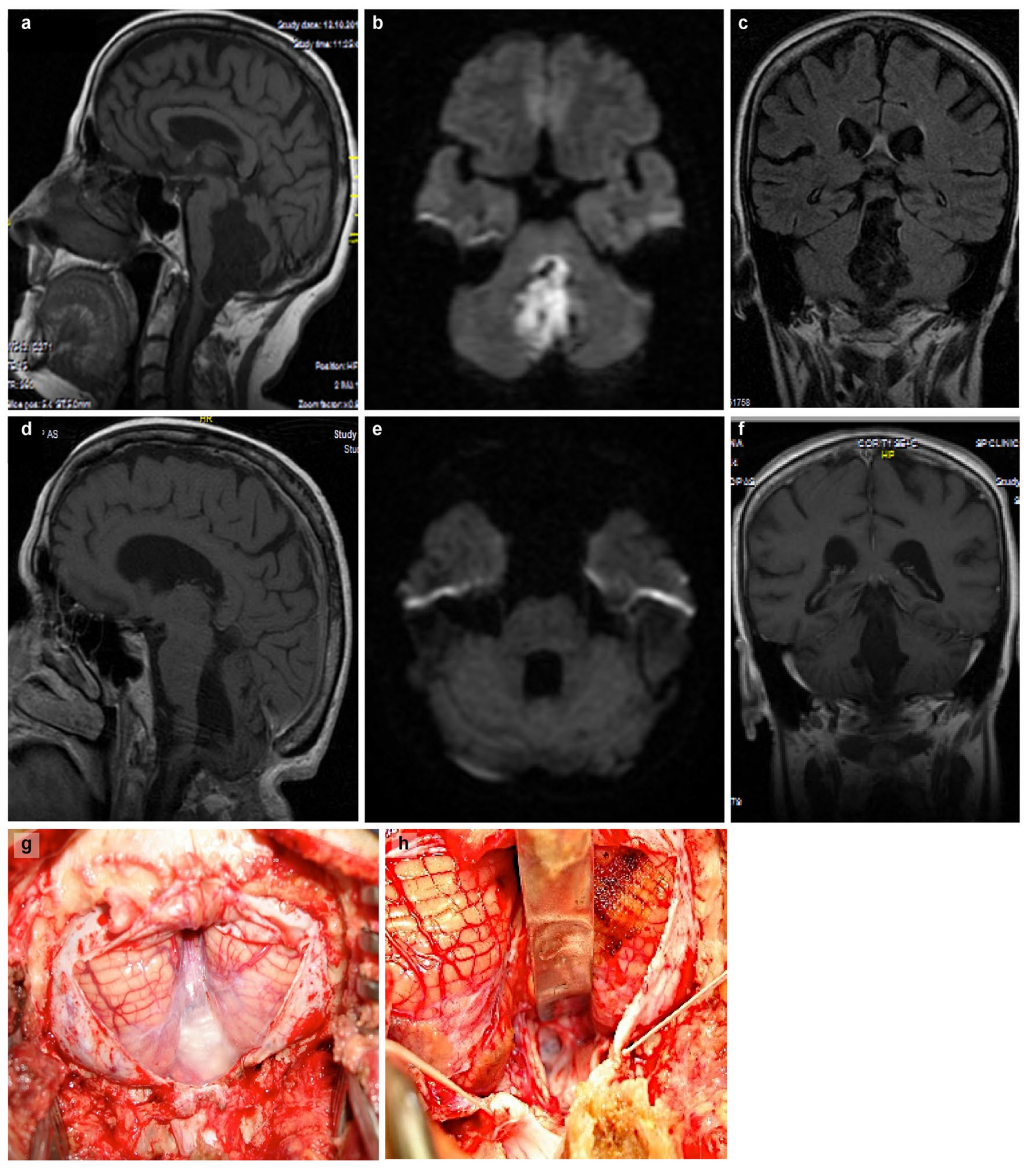
Patient 1, IVth ventricle epidermoid cyst**: **images (**a**–**c**)—preoperative MRI (T1 DWI in sagittal, axial and coronal sequences) revealed a hypodense mass, respectively hyperdense in DWI, filling the fourth ventricle; 3-month follow-up MRI: images (**d**–**f**) (T1 and DWI sequences) demonstrate gross-total resection without remanence at the level of the fourth ventricle floor; images (**g**) and (**h**)—intraoperative aspect, median suboccipital approach.

**Figure 3:**
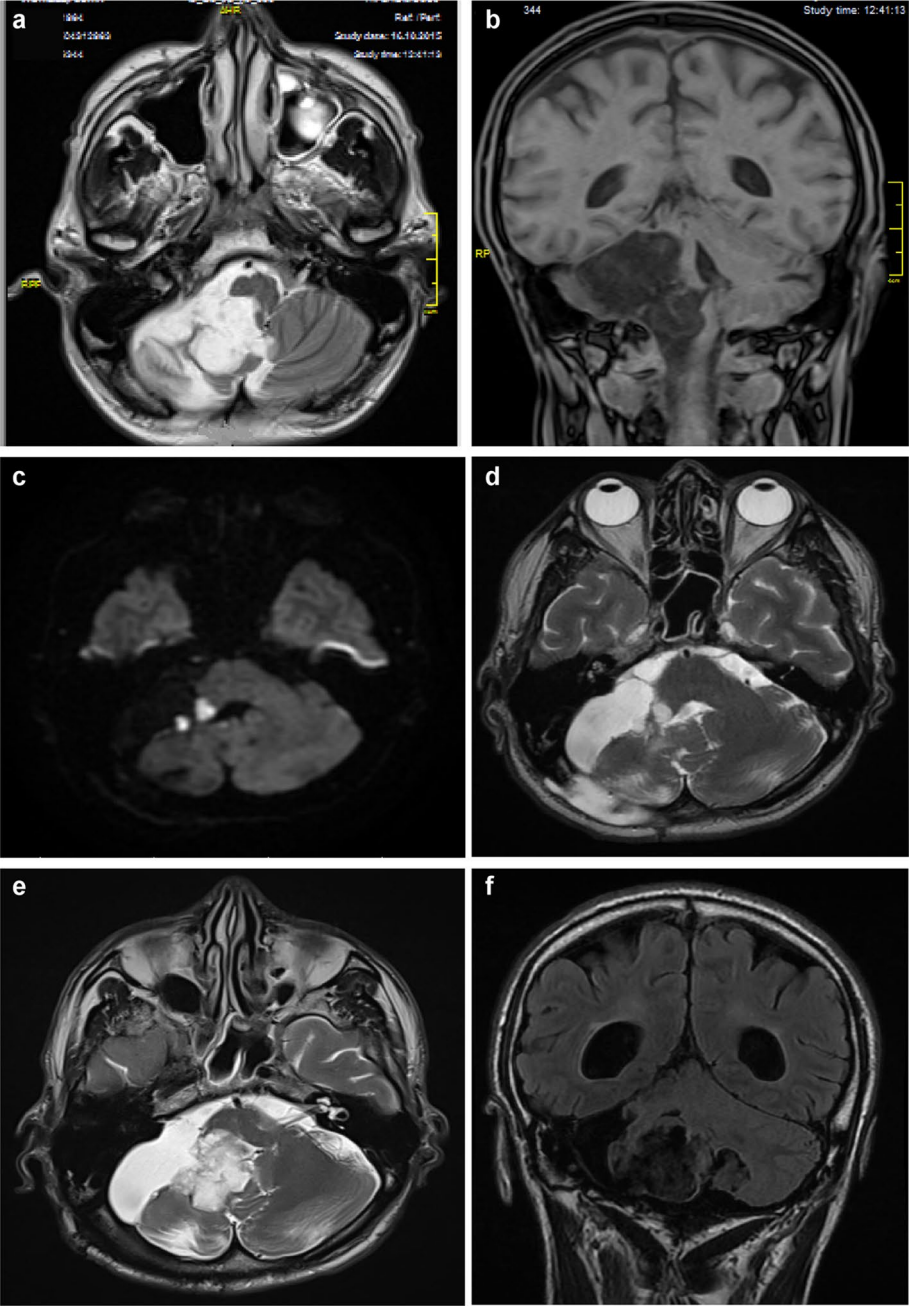
Patient 2, recurrent cerebellopontine angle epidermoid cyst, preoperative MRI (performed in 2015). Images (**a**) and (**b**) T2 and T1 sequences, respectively, revealed an epidermoid cyst located in the right cerebellopontine angle. Three-month follow-up MRI (images **c** and **d**—DWI sequence and T2 sequences, respectively) demonstrated a subtotal resection with a small remnant deeply located in the right middle cerebellar peduncle. The patient was asymptomatic, so he was monitored periodically with MRI imaging at 6-month intervals. The last MRI performed in 2022 demonstrated a large recurrent tumoral mass, but the patient was neurologically stable and asked for delayed reintervention (image **e**—T2 sequence and image **f**—T1 sequence).

In 30 cases, gross total resection was feasible, while in the remaining patients, only subtotal resection was achieved. The most commonly used surgical approaches included retrosigmoid (36.1%), subfrontal (19.4%), telovelar (19.4%) or other (25.1%) (e.g., frontal, subtemporal, transcortical, lateral orbital approach, combined sylvian and retrosigmoid) approaches. In 88.9% of cases, an improvement in symptoms was observed with an average Glasgow Outcome Scale (GOS) value of 4.61. In our study, gross total resection was feasible in 30 cases (83.3%) (Fig. 2). Tumor recurrence was observed in 22.2% (8 cases) of patients in both subtotal and total resection cases (Fig. 3), with a median time to recurrence of 7.5 years. The follow-up period was between 6 months and 10 years. Regarding the resection status of the 8 patients who had recurrence, 5 of them benefited from gross total resection at the first intervention, while 3 of them benefited from subtotal resection. Preoperative hydrocephalus was observed in 37.5% of patients in the recurrence group, while postoperative hydrocephalus was presented by 50% of patients in the recurrence group.

While the distribution between the median and paramedian cisterns was similar in our cohort (Table 1), several clinical parameters correlated with tumor localization. Localization to the median cisterns was associated with hydrocephalus at presentation, protracted hospitalization, and incidence of postoperative hydrocephalus (Table 2). Further multivariate analysis revealed that postoperative hydrocephalus and age < 40 years were prognostic factors for the presence of long-term tumor recurrence (Table 3).

**Table 1:** Distribution of the patients according to the tumor location.

Location	Nr.	Percentage (%)
Median line	15	41.6
Paramedian location	17	47.2
Outside cisterns	4	11.2

**Table 2:** Correlation between the tumor location and different variables.

Variables	Median/Paramedian/Outside Cisterns (p*)
Age (< 40 years, ≥ 40 years)	0.068
Onset of symptoms (insidious, suddenly)	0.734
Duration of symptoms (< 1 month, 1 month–1 year, > 1 year)	0.856
Gender (male, female)	1.000
Hydrocephalus on first presentation (absent, present)	0.005
Duration of hospitalization (< 10 days, ≥ 10 days)	0.032
Postoperative hydrocephalus (absent, present)	0.046

**Table 3:** Correlation between tumor recurrence and different variables.

Variables	Recurrence (absent, present) (p*)
Median/paramedian/outside cisterns	0.734
Degree of removal	0.109
Postoperative hydrocephalus	0.030
	Recurrence (< 5 years, ≥ 5 years)
Age (< 40 years, ≥ 40 years)	0.013

## Discussion

In the surgical experience of the main author (I. St. Florian) over the last 10 years, epidermoid cysts represent 0.85% (36 out of 4233) of operated brain tumors. Despite their benign nature, intracranial epidermoid tumors can be challenging to manage due to their tendency to adhere to critical neurovascular structures, forcing conservative management. Failure to remove the entire proliferative capsule of the tumor augments recurrence rates sevenfold (to 21%), as was reported by Shear et al. in a 691-patient meta-analysis^19^. In unresectable or recurrent cases, adjuvant radiotherapy may be pertinent^20^. Furthermore, adhesions and scar tissue significantly hinder reoperation success rates, which have been reported to be as low as 16.7% versus 73% in de novo cases^2,19^.

In our study, the median age at presentation was 40.4 years with a 1.2:1 predilection to affect male versus female patients, which is on par with other large case series reported in the literature^2,19,21,22^ where epidermoid cysts generally become symptomatic during the fourth decade of life. Symptoms are driven by the compression of local structures and are based on tumor location—in our cohort, chronic intractable headache, dizziness, and cranial nerve palsies were the most common signs. The median symptom duration was 90 days, with minimum and maximum values of 4 days and 4 years, respectively. Stratifying patients into younger (< 40 years, n = 13) and older (≥ 40 years, n = 23) cohorts revealed differences in acute versus chronic symptom presentation. In younger patients, symptom onset was acute in 38.46% of cases, in which the symptoms lasted less than one month and all patients showed signs of increased ICP (intracranial pressure) at admission. In older patients, the onset of symptoms was acute in only 13.04% of cases, in which the symptoms lasted less than five months and were caused by mass effect, increased ICP, or both.

A substantial part of the literature links trigeminal neuralgia and hearing impairment with more acute symptom onset^23–26^. However, in our study, trigeminal neuralgia and hearing impairment were present in 5.6% and 19.4% of patients, respectively, in whom the symptom onset was insidious (more than one month).

Rapid symptom onset was also correlated with the presence of hydrocephalus^23^. Hydrocephalus was reported in 16.7% of our patients at admission; half of them had a sudden onset of symptoms (several days), while the other half reported a gradual onset over approximately one month. Among patients with hospitalized hydrocephalus, in 66.66% of cases, the hydrocephalus persisted postoperatively (tetraventricular hydrocephalus—2 cases, triventricular hydrocephalus—2 cases) with the need for CSF diversion procedures in the form of a ventriculoperitoneal shunt in all 4 cases. Additionally, there were three more new cases of tetraventricular hydrocephalus in the postoperative setting, requiring shunting in two cases.

Intracranial epidermoid lesions are most commonly located in the infratentorial region (47.2%), with a predilection for the cerebellopontine angle (38.9%). According to other studies^24,27^, these lesions are the third most common lesions in the cerebellopontine angle after meningiomas and acoustic neurinomas**.** In our study, we found a statistically significant association (*P* = *0.006*) between infratentorial tumor location and the incidence of postoperative complications (78.6%), which was strikingly higher than that for supratentorial tumors (7.1%). This is consistent with similar studies that evaluated the complications of tumors following epidermoid surgery in the posterior fossa^22,28^.

After transient CN palsies, aseptic meningitis is the second most common postoperative complication^19^. This sequela arises secondary to the spilling of irritant material from the tumor into the subarachnoid space—incomplete resection of the capsule can also contribute to this incidence^2^. The incidence of meningitis can be reduced with radical resection^7,23,27,29^. This complication was seen in two patients (8 and 11 years of age) after subtotal resection of the tumor, which improved following lumbar drainage and treatment with dexamethasone for 7 days after surgery. In these two cases, the tumor was located in the cerebellopontine/cerebellomedullary cisterns and in the pontine cistern, causing CN deficits.

Given that the most common location of the tumor is the cerebellopontine angle, the retrosigmoid approach is an important operative approach for epidermoid tumors^1,30^. In our cohort, we used this approach when the tumor was localized to the cerebellopontine, ambient or cerebellomedullary cisterns—lateral suboccipital (retrosigmoid) craniotomy was performed with patients placed in the sitting position. For tumors located in the fourth ventricle, quadrigeminal cistern and cisterna magna, we performed a median suboccipital craniotomy with patients placed in the sitting position. In the current series, we documented one case of pulmonary embolus arising secondary to the use of the sitting position. In cases where median suboccipital craniotomy was chosen, two patients developed subdural hygroma and pseudomeningocele, which were successfully managed by lumbar drainage.

In 19.4% of cases, a pterional approach or a more limited frontolateral approach was used to reach tumors in the chiasmatic, suprasellar and carotid cisterns. Although postoperative seizures are more common after tumor removal via the subtemporal approach^28,31^, we nonetheless encountered one case of transient postoperative seizures and acute subdural hematoma after using the subfrontal route to remove an epidermoid tumor from the chiasmatic cistern. The frontal paramedian approach was used for tumors located in the pericallosal cistern and subarachnoid space, while the subtemporal approach was used to reach tumors located in the ambiens and interpeduncular cisterns.

As a general neuro-oncological theme, gross total resection offers definitive treatment and drastically mitigates tumor recurrence risk. This concept is especially relevant for intracranial epidermoid tumors, where subtotal resection yields overall recurrence rates of up to 22%^19^. Furthermore, a pooled analysis of four case series (n = 71) found no statistically significant differences in transient or permanent complication rates between gross total and subtotal resection. While the resection margin should be carefully balanced against precipitating iatrogenic neurologic deficits, when subtotal resection is not feasible, it can still portend complication rates that are on par with gross total resection^2,14,27,32^. Preoperative capsular enhancement, diameter > 4.5 cm, calcification, and multicompartmental distribution have previously been identified as predictors for tumor recurrence^13^. In the case of subtotal resection, capsule fragments adherent to neurovascular structures should be devitalized to reduce the risk of recurrence^33^. In our study, gross total resection was feasible in 30 cases (83.3%). Tumor recurrence was observed in 22.2% of patients and was reported in both subtotal and gross total resection cases, with a median time to recurrence of 7.5 years. In the recurrent cases, we did not find any instances of malignant transformation.

The most common permanent postoperative complication in the literature, as well as in our cohort, is hydrocephalus that required shunting^13,34^. To appraise the connection between anatomical localization and the incidence of pre- and postoperative hydrocephalus in our cohort, we classified and evaluated the tumor distribution by median and paramedian cisterns. All cysts growing from the interpeduncular cistern, pericallosal cistern, suprasellar cistern, chiasmatic cistern, quadrigeminal cistern, 4th ventricle, cisterna magna and cerebellomedullary cistern were classified as median line cysts. Cysts growing from the cerebellopontine cistern, ambient cistern, carotid cistern and Sylvian cistern were classified as paramedian cysts^16–18^. In our study, tumor distribution among the median and paramedian cisterns was relatively close (Table 1), unlike other studies that reported an affinity of epidermoid cysts for paramedian localization^35,36^. Obstructive hydrocephalus is more common in midline tumors, as paramedian tumors are less likely to impinge on CSF circulation pathways^37,38^. However, obstructive hydrocephalus can also be a consequence of repeated bouts of mild aseptic meningitis, where dissemination of cyst contents in the subarachnoid space causes persistent hydrocephalus secondary to scarring of the arachnoid villi^39–42^. In our study, patients with hydrocephalus at first presentation were more likely to have a midline tumor (40% versus 0%, *p* = *0.005*); we also found a positive correlation between tumors located in the median cisterns and postoperative hydrocephalus (85.7% versus 31%, *p* = *0.046*). Midline tumors localized to the fourth ventricle were most likely to present with preoperative hydrocephalus that required postoperative shunting, but there are studies that describe fourth ventricle epidermoid tumors without hydrocephalus^27,39^ or report the disappearance of symptoms after surgery^27,43,44^.

The proposed mechanism in all cases of postoperative communicating hydrocephalus was impaired CSF absorption via chemical meningitis. In cases where the tumor capsule was firmly adhered to surrounding structures, we did not perform dissection or coagulation of this part of the capsule. Chemical meningitis can occur by spillage of the cyst contents during the operation; such cases are usually transient and self-limiting and can be managed successfully with steroids. In our opinion, hydrocephalus is a prognosticator of recurrence (*p* = *0.030*), influencing the patient's outcome (*p* = *0.024*). Despite adequate resection, epidermoid cysts have a predilection to recur because of remnant microscopic disease that is often difficult to remove. Those remnants can serve as a nidus for regrowth via continued desquamation. Fortunately, these cysts do not repopulate with exponential growth but rather have a linear pattern of desquamation that may require patients to have a second surgery later in life when they are symptomatic again. However, this continued desquamation can be responsible for sustained inflammation and subsequent hydrocephalus. In such cases with gross total resection but active microscopic remnants, the appearance of hydrocephalus can precede a recurrence^2,39–45^.

Midline tumor location was also associated with a protracted hospitalization course exceeding 10 days (86.7% versus 25%, *p* = *0.032*), which is expected given the higher pre- and postoperative symptom burden in these patients. Approximately half of the patients who were admitted for longer than 10 days also had, on average, at least one postoperative complication. Precipitation of symptoms (under 1 month) was associated with a higher rate of postoperative complications (p = 0.026); no association was found between long-standing symptomatic patients and the rate of postoperative complications (p = 0.44). There was no statistically significant association between tumor location and sex *(p* = *1.000*), patient age (*p* = *0.068*), acute or chronic symptom onset *(p* = *0.734*), or symptom duration (*p* = *0.856*). However, there was a correlation between age ≥ 40 years and paramedian tumor localization (82.4% versus 46.7%), and vice versa—age < 40 years and median tumor localization (53.3% versus 17.6%). This disparity might be explained by the higher incidence of obstructive hydrocephalus in median tumors, where smaller tumor volumes are required to precipitate acute symptoms secondary to impeding CSF outflow. In contrast, paramedian tumors have a longer latent period of growth, where compressed and/or atrophied normal adjacent brain tissue can accommodate larger tumor volumes without the presence of critical symptoms^37,38^.

Multivariate analysis suggested that median versus paramedian localization had no influence on recurrence rates *(p* = *0.734*). Although the extent of resection is a well-established prognostic factor for recurrence, this criterion did not reach statistical significance in our study (*p* = *0.109*), most likely due to the small sample size. Rather, the incidence of postoperative hydrocephalus was strongly associated with recurrence (50% vs. 10.7%, *p* = *0.030*)—this was especially relevant in patients < 40 years of age, in whom the recurrence interval was lower than that in patients ≥ 40 years of age (2.67 ± 1.52 years vs. 11.2 ± 3.96 years, *p* = *0.013*). In our study, recurrence rates in patients with subtotal resection were approximately 50% within a follow-up period of five years, which is consistent with previously reported studies^41–43^.

## Conclusions

Despite their benign biology, primary intracranial epidermoid tumors are challenging clinical entities due to their recurrence statistics, lack of systemic treatment options, and high rates of postoperative complications even in patients with more sparing subtotal resection.

To our knowledge, this is the first study in the literature to evaluate the relationship between epidermoid cysts and median versus paramedian localization. For median epidermoid cysts discovered accidentally or with minor symptoms in younger patients, major attention should be given due to the increased risk of developing hydrocephalus, which leads to a higher degree of tumor recurrence in less than 5 years after surgery. For these patients, from our point of view, both the neurologist and the neurosurgeon who are following the patient should reevaluate the patient as often as possible to raise the issue of surgical treatment as soon as possible.

## Data Availability

The datasets analyzed during the current study are available from the corresponding author on reasonable request.
